# Case report: Suspected plasmablastic lymphoma in a dog resembling the clinical presentation in humans

**DOI:** 10.3389/fvets.2023.1100942

**Published:** 2023-02-16

**Authors:** Antonio Giuliano, Sin Yan Lam, Mayra C. Flecher, Rodrigo S. Horta

**Affiliations:** ^1^CityU Veterinary Medical Centre, City University of Hong Kong, Kowloon, Hong Kong SAR, China; ^2^Department of Veterinary Clinical Sciences, Jockey Club College of Veterinary Medicine, City University of Hong Kong, Kowloon, Hong Kong SAR, China; ^3^Veterinary Emergency Center, Kennedy Town, Hong Kong SAR, China; ^4^Department of Veterinary Medicine, Universidade Vila Velha, Vila Velha, ES, Brazil; ^5^Department of Veterinary Medicine and Surgery, Veterinary School, Universidade Federal de Minas Gerais, Belo Horizonte, MG, Brazil

**Keywords:** plasma cells, B-cell, neck mass, lymphoma, immunohistochemistry

## Abstract

Plasmablastic lymphoma (PBL) is a rare form of lymphoma in people. PBL originates from plasmablasts and usually presents with swelling/mass in the mouth/neck. A 7-year-old Mongrel dog was presented for a large oral and neck mass. Cytology and histopathology were suggestive of a round cell tumor that was suspected to be lymphoma. An immunohistochemical (IHC) stain panel showed positive for CD18, thus supporting the diagnosis of round cell tumor, but negative for T- and B-cell lymphomas, CD3, CD20, and PAX-5. Other markers including cytokeratin AE1/3 (for epithelial cell origin), CD31 (for endothelial cells), SOX10 (for melanoma), IBa-1 (for histiocytic sarcoma), and CD117 (for mast cell tumor) were all negative. MUM-1 (for plasma cell differentiation) was strongly positive and CD79a (B cell and plasma cells) was also scantly positive. Based on the histopathology and immunohistochemistry results in combination with the clinical presentation, a suspected diagnosis of PBL was made. As per available literature, this is perhaps the first highly suspected case of PBL in a dog.

## 1. Introduction

Plasmablastic lymphoma (PBL) is a rare type of B-cell lymphoma that is more commonly seen in people with HIV (1). While PBL has been documented in people without HIV, immune deficiency seems to have a significant role in the pathogenesis of this type of lymphomas. Often, people who develop PBL have a history of immunosuppression, such as the use of prednisolone, organ transplant, or concurrent cancer (2–4).

PBL is an aggressive form of large B-cell lymphoma that originates from plasmablasts that have undergone somatic mutations during differentiation from centrocytes to mature plasma cells (1). PBL could be considered a rare and aggressive form of the more common plasmacytic/plasmacytoid lymphoma that usually has a less-aggressive behavior (5). The PBL immunophenotype in people shows positive markers for plasma cells (CD79a, IRF-4/MUM-1, BLIMP-1, CD38, and CD138) and negative B-cell markers (CD19, CD20, and PAX-5) (6).

The final diagnosis can be difficult as plasmablastic or anaplastic multiple myeloma may be morphologically and immunophenotypically identical to PBL (6).

Although PBL has a heterogeneous clinical presentation in both patients who tested positive and those who tested negative for HIV, it often presents with an oral mass and/or a mass that extends next to the oral cavity like the mandible and neck (1). Any organ can be affected such as lymph nodes, skin, gastrointestinal tract, bone, and musculature (7). In people, PBL has an aggressive clinical course with a very poor prognosis (7). Response to chemotherapy is also quite poor with only 45% of patients achieving a complete response (8).

While the prognosis for untreated patients is only 3–4 months, people treated with CHOP-based protocol (doxorubicin, vincristine, cyclophosphamide, and prednisolone) survive for 15 months (3, 9). There is no standard of care for the treatment of PBL, and even chemotherapy protocols with more intense doses/schedules than the standard CHOP protocol cannot achieve any survival advantage (8).

The WHO classification of lymphomas for humans has also been used for canine lymphoma. In this classification, plasmacytic lymphoma is considered a rare type of lymphoma, while plasmablastic lymphoma cases have not been reported previously (10). In a more recent retrospective study of 203 canine multicentric lymphomas, lymphomas with plasmacytic or Mott cell differentiation corresponded to approximately 2% of all the cases, but again no PBL cases were reported (11). In the literature, there are also occasional case reports of lymphoma with plasmacytic or Mott cell differentiation with the involvement of skin (12), gastrointestinal tract and abdominal lymph nodes (13, 14), liver and spleen (15), and multicentric lymphomas (16, 17), including a case report with confirmed leukemia with plasmablastic differentiation (18). To the best of the authors' knowledge, previous cases of PBL with presentations similar to human PBL have never been reported. This can be due to underreporting and lack of awareness of the presence of this specific type of lymphoma or difficulty in achieving a final diagnosis. The diagnosis of plasmacytic lymphoma is based on the plasmacytoid and/or Mott cell differentiation morphology and is confirmed by positive immunoreactivity for CD20, CD79a, and/or MUM-1 (15, 17, 18). However, a definitive diagnosis of PBL in dogs is difficult due to the lack of typical histological appearance and specific immunohistochemistry stains that are available for human PBL. To the best of the authors' knowledge, this is the first case report of suspected PBL in a dog, with the clinical presentation resembling its human counterpart.

## 2. Case report

A 7-year-old female spayed mixed-breed dog presented for further investigation of a mass on the right side of the mandible and neck. The dog had a history of ehrlichiosis that was successfully treated 2 years before the presentation. The dog was also treated for atopic dermatitis with corticosteroids and then for pruritus with the monoclonal CD31 antibody lokivetmab (Cytopoint^®^, Zoetis).

During the presentation at the referring veterinarian, a 5 cm mass was present at the right side of the neck (suspected to be a retropharyngeal/mandibular lymph node enlargement). A hard-fixed non-well-defined mass was also present on the left side of the tongue/mandible. Physical examination was otherwise unremarkable, and no other lesions were noticed. The dog was otherwise bright, alert, and responsive and no other concerns were reported by the owner. Hematology and biochemistry, including electrolytes and total calcium, were unremarkable. No cytopenia, hypercalcemia, or hyperglobulinemia were present. Fine-needle aspiration of the mass in the neck was also performed. The cytology report from a certified clinical pathologist was highly suspicious of lymphoma, but other round cell neoplasia could not be ruled out. Due to the unusual presentation, a decision was made to perform a biopsy.

Incisional biopsies of the oral and neck mass were performed, and histopathological analyses were suggestive of a poorly differentiated round cell neoplasia with lymphoma being the most likely differential diagnosis. An immunohistochemical (IHC) panel showed positive for CD18, supporting the diagnosis of round cell tumor but negative for T- and B-cell lymphomas, CD3, CD20, and PAX-5. IHC stains, including SOX10, IBa-1, and CD117, showed no immunoreactivity that ruled out the presence of melanoma, histiocytic sarcoma, and mast cell tumors. A non-B non-T cell or NK lymphoma was suspected by the pathologist.

**Figure 1:**
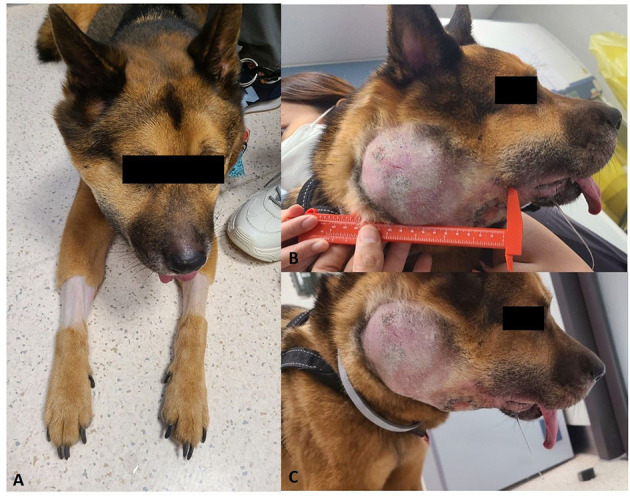
Clinical presentation of the dog with suspected PBL. **(A)** Cervical mass on the right side. **(B, C)** Right oral/neck mass was very large (approximately 15 × 10 cm).

Conventional treatments for lymphoma were declined by the owner and the dog received only acupuncture treatment. After 2 months, the dog was represented due to a marked progression of the disease. On presentation, the dog was alert and responsive but was underweight (3/9 body condition score). Vital parameters were within normal limits, and thoracic auscultation and abdominal palpation were unremarkable. The right oral/neck swelling/mass was very large (approximately 15 × 10 cm), hard, and fixed on palpation. In some ventral areas of the mass, ulceration and necrosis were also present. The right side of the face was significantly swollen and a clear distinction between the oral mass and neck mass was difficult (Figures 1A–C).

The right mandibular lymph node was not palpated, while the left was normal in size. While both prescapular lymph nodes were slightly enlarged, the popliteal nodes were normal in size and the inguinal and axillary lymph nodes were not palpable. Multiple ill-defined subcutaneous/muscular masses were palpable upon physical examination at different locations, including the left flank and the left ventral abdomen.

**Figure 2:**
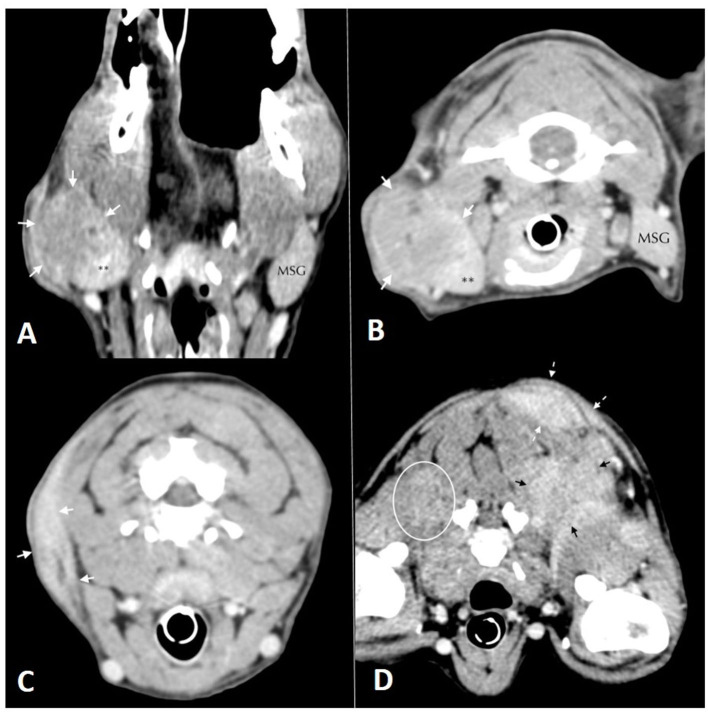
Soft tissue window computed tomographic images. **(A)** (left) and **(B)** (right): in **(A)** dorsal and **(B)** transversal planes oriented with the patient's right side on the left of the image. Mass lesion (white arrows) arising from the lateral margin of the right mandibular salivary gland**. The left mandibular salivary gland (MSG) is labeled for comparison. **(C)** (left), **(D)** (right): Soft tissue window computed tomographic images in the transverse plane, oriented with the patient's right side on the left of the image. Images show multiple mass lesions arising from the **(C)** subcutaneous tissues of the right aspect of the neck (white arrows), **(D)** poorly defined hyper-contrast enhancing mass lesions in the musculature deep to the scapulae bilaterally (white oval and black arrows) and more well-defined mass lesions within the dorsal, left subcutaneous tissues (dashed white arrows).

A full body CT scan showed a large mass in the neck region arising from the subcutaneous tissues of the right neck at the level of the lateral margin of the right salivary gland. The mandibular and retropharyngeal lymph nodes were normal in size (Figures 2A, B). Other masses were present on the left longissimus/iliocostalis and right longissimus muscles, the caudal margin of the right antebrachium, the left body wall at the level of L2, and the left caudal ventral body wall. Thoracic and abdominal CT were unremarkable aside from mildly enlarged left axillary and external iliac lymph nodes. No osteolytic lesions were noticed in any of the bones (Figures 2C, D).

**Figure 3:**
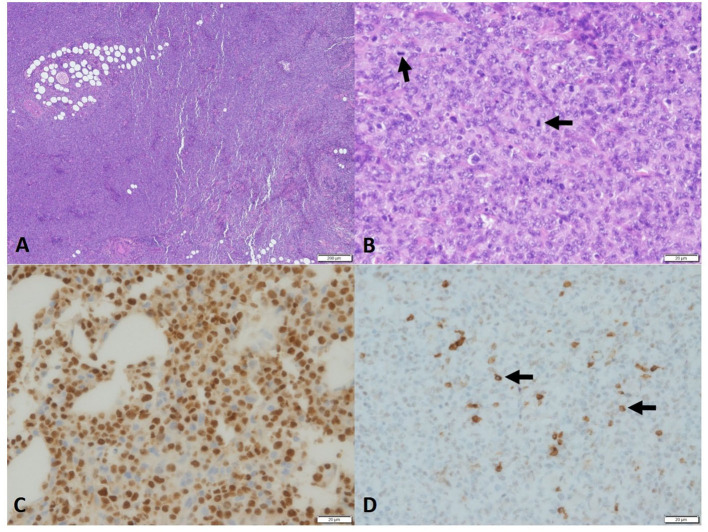
Photomicrograph of the sample of PBL in the dog. Histopathology of neoplasm, **(A)** high cellularity, no encapsulation, and infiltration. 4×, H&E. **(B)** Round cells, eosinophilic cytoplasm, oval nucleus, and eventually reniform. Chromatin-dispersed single central nucleolus and marked anisokaryosis. Many mitosis figures (arrow), 40× H&E. Immunohistochemistry, **(C)** MUM1, Neoplastic cells showed strong nuclear staining. **(D)** CD79 immunohistochemistry. Some neoplastic cells, 40× (< 20%) showed strong cytoplasmic staining, 40×.

Due to the unusual presentation of lymphoma, multiple incisional biopsies were performed at the right neck/submandibular and left body wall masses. The histopathological examination of a second pathologist confirmed the previous diagnosis of a poorly differentiated round cell neoplasia, characterized by dense neoplastic proliferation infiltrating adjacent collagenous and adipose tissues. Neoplastic cells were rounded with often indistinct cytoplasmic borders, clear eosinophilic cytoplasm, oval to reniform and sometimes eccentric nuclei, dispersed to condensed chromatin, single central nucleolus, marked anisokaryosis, moderate nuclear pleomorphism, and 40–60 mitotic figures in 10 400 × fields (2.37 mm^2^). No difference in microscopic appearance from the previous histopathology was reported (Figures 3A, B).

Further IHC stains, including cytokeratin AE1/3 (for epithelial cell origin) and CD31 (for endothelial cells), were all negative. MUM-1 (for plasma cell differentiation) staining was positive with moderate-to-strong immunoreactivity and approximately 90% of positive neoplastic cells, and CD79a was also scantly positive (Figures 3C, D).

A diagnosis of poorly differentiated anaplastic multiple myeloma vs. plasmablastic lymphoma was suspected based on the histopathological round cell/lymphoma appearance, IHC positivity to MUM-1, and weak positivity to CD79a, while all the other IHC markers investigated were negative. A diagnosis of solitary extramedullary plasmacytoma was considered very unlikely due to the aggressive and rapid progression of the tumor and the multifocal involvement of various subcutaneous tissues and muscles. This clinical presentation was similar to that of PBL in people where an oral (often lateral mandible) mass extending to the neck is usually the most common first presentation followed by the rapid dissemination of the neoplasia throughout the body.

The dog was started on a modified CHOP plus asparaginase protocol, but only a very short partial response to vincristine and no response to asparaginase, doxorubicin, and cyclophosphamide was achieved. The dog progressed very rapidly despite treatment and died 3 months post-diagnosis. A post-mortem examination was declined by the owner. The rapid progression, poor response to chemotherapy, and very poor prognosis were also comparable to the human PBL presentation.

## 3. Discussion

This is the first case described of suspected PBL in a dog with clinical presentation similar to the human counterpart. Despite the difficulties in differentiating PBL from undifferentiated or anaplastic multiple myeloma based on both histopathology and immunophenotypes, the clinical presentation, laboratory findings, and the very rapid and aggressive course of the disease were more consistent with PBL in humans.

In addition, in humans, the final diagnosis of PBL can be very difficult as plasmablastic or anaplastic multiple myeloma can be morphologically and immunophenotypically identical to PBL (6). PBL in humans is an aggressive form of large B-cell lymphoma that exhibits plasmablastic morphology (1). The cells of origin of PBL are believed to be the plasmablasts, activated B-cell centrocytes that transform into plasmablasts before becoming mature plasma cells (7). Phenotypically, plasma blast cells express CD38 and multiple myeloma oncogene 1/interferon regulatory factor 4/(MUM-1/IRF-4) and lose CD20, while maintaining CD19 expression (7, 19). The PBL immunophenotype in humans shows positive markers for plasma cells (CD79a, IRF-4/MUM-1, BLIMP-1, CD38, and CD138) and negative for B-cell markers (CD19, CD20, and PAX-5) (6). Unfortunately, in dogs, not all the markers are available for a more detailed phenotype assessment, and the diagnosis of PBL was mainly based on the cytological and histological appearances, the positive result to MUM-1 that confirms the plasma cell origin, and the combination of clinical presentation and a rapid and aggressive clinical course. In this case, CD79a was only weakly positive. However, even in humans, only 45% of the PBL cases are positive for CD79a and loss of CD79a in B-cell lymphoma and multiple myeloma (MM) is not uncommon, especially in poorly differentiated tumors (20).

Lymphomas with plasmacytic/plasmacytoid/Mott differentiation appearance have been previously reported in dogs. Snyman et al. (17) describe a case of multicentric plasmacytic lymphoma in an Australian Shepherd with the infiltration of lymph nodes, tonsils, gastrointestinal tract, spleen, kidneys, lungs, and brain. Neoplastic cells were positive for CD79a, MUM-1/IRF-4, LLC (lambda light chain), and CD18 but negative for CD3 (17). A 5-year-old Golden Retriever was diagnosed with plasmablastic leukemia by positive immunolabeling for MUM-1/IRF-4 but negative for CD204, Iba-1, E-cadherin, CD3, CD5, CD79a, CD20, and PAX5 (18). In one dog with non-epitheliothropic plasmacytic lymphoma, the tumor cells presented positive immunoreactivity for CD79a and MUM-1/IRF-4 but negative immunoreactivity for CD20, PAX5, CD3, and Iba1 (12).

Besides, the dog in our case report presented similar clinical findings to humans with PBL, often described as the presence of an oral mass that rapidly progresses with the invasion of the neck region followed by the involvement of various organs. The clinical presentation in this dog was extremely unusual for both lymphoma and multiple myeloma (MM). Despite the MUM-1 positivity that suggests a plasma cell origin, no clinical findings suggestive of multiple myeloma were present, including a lack of osteolytic lesions, cytopenia, hypercalcemia, or increased globulins. Despite this, a bone marrow biopsy was not performed to rule out initial BM involvement as MM remains unlikely due to its usually slow clinical progression, which is very different from the clinical presentation and rapid deterioration seen in this case. Even if this is considered unlikely, we cannot completely rule out another type of undifferentiated round-cell neoplasia with aberrant MUM-1 expression.

In this highly suspected case of PBL, such as in humans, the response to chemotherapy was poor and short lived and the dog survived only for 3 months despite CHOP chemotherapy treatment.

However, this type of lymphoma in humans has been associated with immunosuppression and prednisolone treatment. The dog in this case had a history of atopy/skin allergy that is believed to be associated with immune dysregulation, in combination with a history of previous treatment with prednisolone that causes systemic immunosuppression (21).

Various spontaneous cancers in dogs are considered useful translational models for human cancer research (22). Lymphoma in dogs, especially the most common type called diffuse large B cell lymphoma (DLBCL), has been considered a useful comparative model for translational research (23).

Lymphoma in people and dogs have similar presentation, response to treatment, and even outcome (5-year survival in people treated with CHOP translates to 1 year in dogs) (22, 24). Interestingly, PBL is a very rare form of lymphoma in people and this has never been described in dogs. Prognosis in people treated with CHOP chemotherapy is very poor with only 15-month survival compared to 5 years of the most common forms of lymphoma. Similarly, in this case of suspected PBL, survival was only 3 months, which is much poorer than DLBCL survival which is approximately 1 year (24).

The prognosis for the previously reported forms of plasmacytic/Mott lymphoma is largely unknown in dogs as only a few cases have been documented. A dog with non-epitheliothropic plasmacytic lymphoma presenting multiple lesions was treated with COP (cyclophosphamide, vincristine, and prednisolone) chemotherapy (only induction phase), resulting in a disease-free interval of 120 days. Relapse was treated with the maintenance of the COP protocol with a second remission longer than 100 days (12). Gastrointestinal presentation of Mott lymphoma resulted in the survival of 3 months in a 2-year-old Dachshund treated with CHOP chemotherapy (14) but longer than 10 months in a 1-year-old Dachshund treated with surgery and COP chemotherapy (13). A 9-year-old mixed breed dog was diagnosed with Mott lymphoma in the liver and the spleen and treated with CHOP chemotherapy and, after relapse, with lomustine and L-asparaginase, resulting in a survival of 5 months (15). A dog diagnosed with plasma-cell leukemia with plasmablastic morphology was treated with a combination of melphalan and prednisolone, but the disease progressed and the dog was euthanized after 15 days (18).

To the best of the authors' knowledge, the case reported is the first PBL case described in a dog and highlights once again the similarity of lymphoma and lymphoproliferative disease in dogs and humans. Veterinarians should be aware of this rare type of highly aggressive lymphoma in dogs.

## Data availability statement

The original contributions presented in the study are included in the article/supplementary material, further inquiries can be directed to the corresponding author.

## Ethics statement

Ethical review and approval was not required for the animal study because this is a retrospective case report. Consent form was obtained before treatment of the dog. The owner gave consent for publication as well.

## Author contributions

AG did primary case management and wrote and reviewed the manuscript. SL did primary case management and reviewed the manuscript. MF reviewed and described the histopathology and immunohistochemistry and reviewed the manuscript. RH wrote and reviewed the manuscript. All authors critically reviewed and approved the final version of the manuscript.
